# Management of neuropathic bladder secondary to spina bifida: Twenty years' experience with a conservative approach

**DOI:** 10.3389/fped.2022.913078

**Published:** 2022-07-29

**Authors:** Hesham Elagami, Tariq O. Abbas, Kathryn Evans, Feilim Murphy

**Affiliations:** ^1^St George's Hospital, London, United Kingdom; ^2^Weill Cornell Medicine - Qatar, Ar-Rayyan, Qatar; ^3^Sidra Medicine, Doha, Qatar

**Keywords:** spina bifida, neuropathic bladder, urodynamics, intermittent catheterisation, conservative, management

## Abstract

**Introduction:**

Treatment of neuropathic bladder secondary to spina bifida is an ongoing challenge. Although different management strategies and protocols are available in the literature, reliance on expert opinion remains fundamental. A conservative approach can be utilized, but patients must be closely monitored throughout the management process. The objective of this study was to review the management and outcomes of neuropathic bladder in spina bifida by appraising long-term bladder and renal function in patients treated at a medical center utilizing a conservative management style.

**Methods:**

This is a single-center retrospective review of urology care for all spina bifida patients 5–19 years of age with a neuropathic bladder who attended follow-ups between April 2000 and April 2020. Only patients with more than 5 years of follow-up were included. Renal functions, continence and results of invasive video urodynamics (IUD) and any surgical interventions were recorded.

**Results:**

Seventy-one patients (mean age = 10.5 years) were identified after exclusions. Bladder compliance between first and last IUDs increased significantly (*p* = 0.0056). Anticholinergic treatment was started at the first outpatient appointment. Intravesical botulinum toxin injection was the second line treatment in ten patients. 94% of patients had an end fill pressure below 40 cm H_2_O in their last IUD. 82% were socially continent (dry or occasional damp patches) with or without catheterisations at the age of 11.5 years. One patient in the cohort had bladder augmentation.

**Conclusion:**

The optimal management of neuropathic bladder secondary to spina bifida remains controversial. Bladder and renal functional outcomes can be improved with close monitoring and less invasive management.

## Introduction

A neuropathic bladder (NB) secondary to spina bifida, if left untreated, can lead to severe renal damage and marked upper tract dysfunction (1, 2). The literature describes many widely varying strategies for management of this condition, with substantial differences including the timeframes for initiating clean intermittent catheterisation (CIC) and anticholinergics and the indications of botulinum toxin injection and ileocystoplasty. This adds to the challenge of treating these patients and reflects the difficulty of anticipating the behavior of bladder development and any subsequent treatment required. CIC and anticholinergics have long been established therapies for NB, aiming to improve continence and bladder safety (3). However, there remains controversy with regards to the frequency of CIC and the best time to initiate it. The British Association of Pediatric Urology consensus discussion in 2016 demonstrated that there is a wide variety in opinions regarding the use and timing of different surgical options for NB (4).

Here, our objective was to review medium- and long-term bladder and renal outcomes in a single center adopting a conservative approach for bladder management. This approach utilized CIC, anticholinergics, botulinum toxin injection and a drainage procedure. Bladder augmentation was indicated if all of the forementioned measures failed. Secondary outcomes included continence, dimercaptosuccinic acid (DMSA) scan results and other related surgical interventions in the final year of follow-up.

## Materials and methods

This is single-center retrospective review of urology care for all spina bifida patients with secondary NB. Patient management began in the neonatal period, with bladder function assessment and start of CIC if indicated by high post-voiding bladder residual. Prophylactic antibiotics were not routinely used for patients on CIC. Table 1 describes this initial protocol. As the aim was to study medium- and long-term outcomes, patients under 5 years of age were excluded. Notes on all patients aged 5–19 years old and managed between April 2000 and April 2020 were reviewed. We included all such patients who were diagnosed with either spina bifida or spinal dysraphism and who had more than 5 years' follow-up. Patients were excluded if they had any other cause for NB, if they were lost to follow-up or if they had had a major bladder intervention prior to being transferred to our unit. Patients' demographics and the levels of their spinal defects were noted. Data were collected from a maintained centralized database of medical records in our hospital, from electronic and paper patient notes and from radiological investigations. These data included DMSA scans, invasive video urodynamics (IUD) results and ultrasound (US) scans. Urodynamics were performed according to the protocol in Table 1. DMSA scans were performed at the age of 3 months, either (a) upon clinical suspicion of renal injury based on US scans or (b) if there was a history of urinary tract infection (UTI).

**Table 1:** Protocol used for the management of spina bifida patients with neuropathic bladder.

**Timing**	**Management plan**
*Neonatal unit*	BFA +/- starting CIC; US renal tract; trimethoprim treatment; neurosurgery and orthopedic input
*6 weeks*	US renal tract and hips; BFA
*3 months*	Spina bifida clinic* and DMSA
*6 months*	Spina bifida clinic – ensure stability and assess bowel
*1 year*	US renal tract; BFA; spina bifida clinic
*18 months*	Spina bifida clinic
*2 years*	Spina bifida clinic; IUD; US renal tract; eGFR
*3–6 years*	Yearly spina bifida clinic reviews, plus: repeat BFA at 4 years repeat IUD with US and eGFR at 6 years
*7–14 years*	Two yearly reviews with BFA, plus: repeat IUD with US and eGFR at 14 years

*BFA, bladder function assessment (non-invasive urodynamics); CIC, clean intermittent catheterisation; DMSA, dimercaptosuccinic acid scan; eGFR, estimated glomerular filtration rate; IUD, invasive video urodynamics; US, ultrasound*.

^*^Joint clinic: urology, neurosurgical and orthopedic.

The evaluation criteria included:

1. Bladder status (safe or unsafe), with a safe bladder defined as a mean detrusor end pressure of <40 cm H_2_O as measured by IUD (5).
a. Maximum bladder capacity – measured by the end fill volume and presented as observed/expected (O/E) volume, using the following formula to calculate expected bladder volume in milliliters: {[patient age (years) + 1] x 30 mL}(6).
b. Detrusor pressure (P_det_) - maximum measured pressure, the end fill volume or the leak point.
c. Presence of detrusor muscle overactivity (DO).
d. Bladder compliance (ΔV/ΔP) (7).
e. Presence of vesicoureteral reflux (VUR).
2. Corrected estimated glomerular filtration rate (eGFR) at last follow-up, to define kidney disease. The radiotracer Tc-99m DTPA was used to measure patients' eGFR, with a diagnostic reference of 10 MBq. This was done according to the protocol in Table 1 at the ages of 2, 6, and 14 years. The height of the patient was measured on the bed (unless this was not physically possible, in which case an estimate was used and the reason for using the estimate was documented). Decreased eGFR was defined as eGFR <90 mL/min/1.73 m^2^ (8).
3. Urinary continence status at 6 years of age and at last follow-up, assessed using patient-completed bladder diaries or by a questionnaire documented in the clinical notes. Social dryness/continence was defined based on the patients' in-clinic reports. Patients were considered “socially continent” if they were (a) consistently dry or (b) occasionally wet with only damp patches (as opposed to soaking their clothes). Patients with occasional damp patches were considered socially continent whether or not they were on CIC. The questionnaire we used in-clinic followed the guidance of the dysfunctional voiding scoring system published by Farhat et al. (9). However, it was not possible to follow the whole published scoring system due to the lack of sensation commonly accompanying SB.

The secondary outcomes included urologically related surgeries and interventions such as drainage procedures and injection of Deflux® or botulinum toxin A (Dysport®). Deflux® was clinically indicated by high-grade (grade 3/4) VUR in an otherwise safe bladder. Botulinum toxin injection was used in cases of high pressure, unsafe bladder and intractable incontinence despite CIC and oxybutynin or alternative antimuscarinic medication. DMSA scan findings were reported when available. Bowel function and fecal incontinence were not considered for this review.

Paired *t*-tests were used to compare IUD outcomes from the first and most recent tests, if available. Fisher's exact test was used to assess categorical data. A *p*-value < 0.05 was considered significant. Values are presented as means (with ranges) unless otherwise specified.

## Results

Ninety-seven spina bifida patients with a diagnosis of NB were identified from our database. Seventy-three were aged over 5 years and were currently under the care of the pediatric urology team. Two of these patients were lost to follow-up or moved outside of our area and were excluded from data analysis. 63% (45/71) of the patients had a lumbosacral myelomeningocele defect and 52% (37/71) were male. Mean age at last follow-up was 10.5 years (range: 5–19).

**Figure 1:**
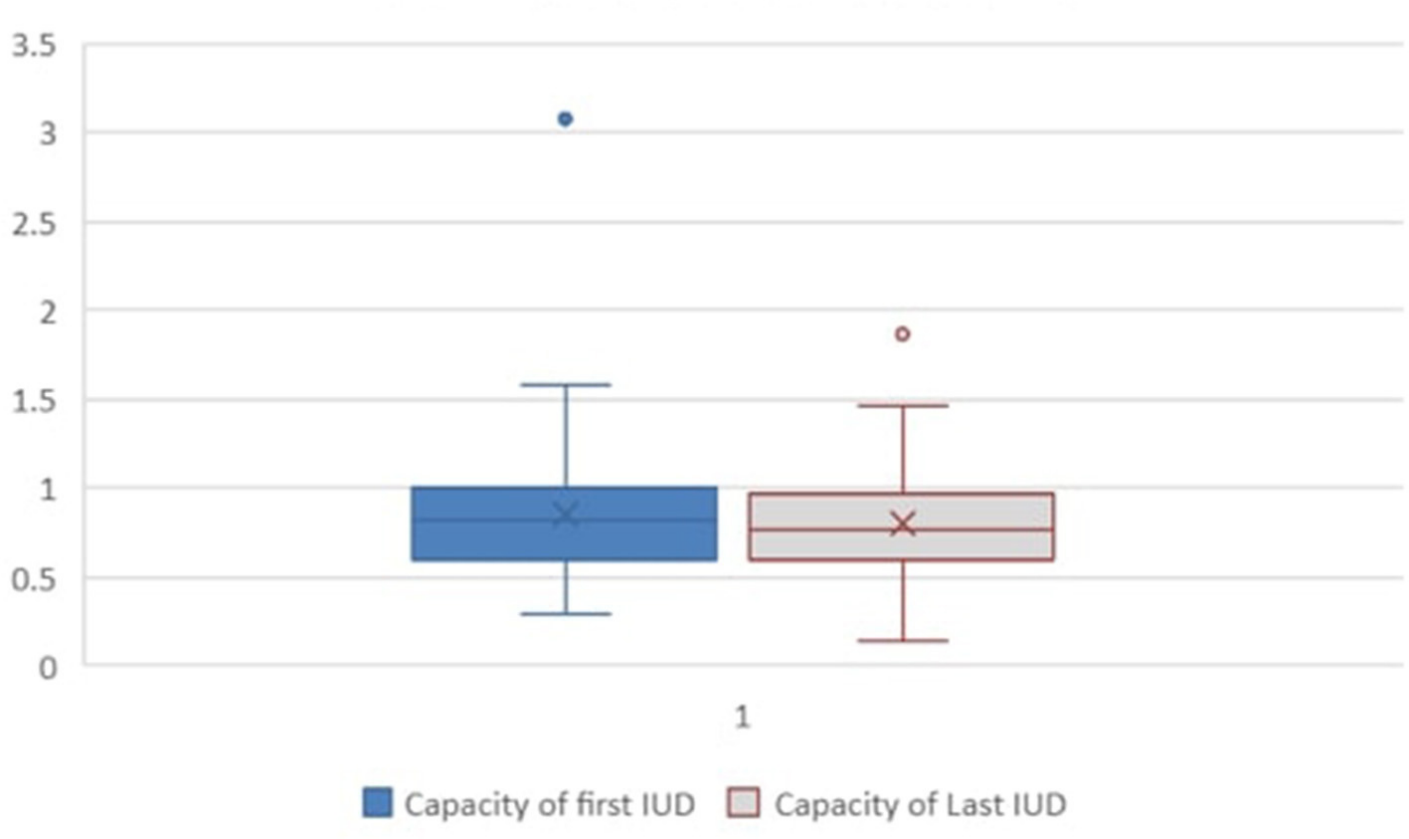
Bladder capacity – observed/expected (O/E) ratio (mL/mL) at first and last recorded IUDs. Fifty-four patients who had more than one test available were included. Statistical significance was assessed using paired *t*-test, and a *p*-value < 0.05 was considered significant. IUD, invasive video urodynamics.

**Figure 2:**
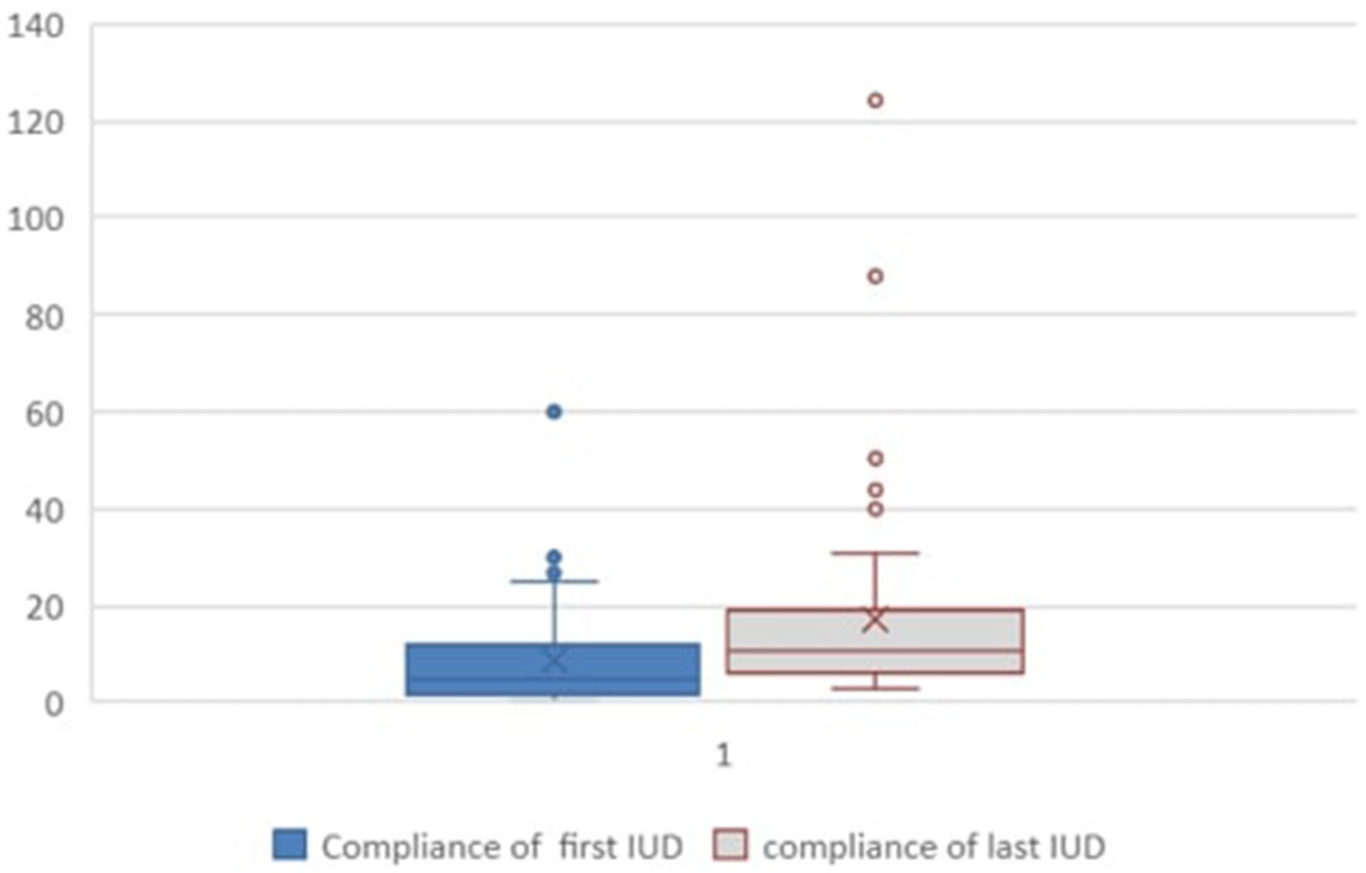
Bladder compliance (mL/cm H_2_O) at first and last recorded IUDs. Fifty-four patients who had more than one test available were included. Statistical significance was assessed using paired *t*-test, and a *p*-value < 0.05 was considered significant. IUD, invasive video urodynamics.

All 71 patients had invasive video urodynamics (IUD) performed at least once during the study period. Fifty-four patients had two or more IUDs available for review. The outcomes from the first and last available IUDs for these 54 patients are presented in Table 2. The observed/expected (O/E) bladder volume ratios on serial investigations had a mean of 0.875 in the initial IUD study and 0.82 in the last one performed (Figure 1). The mean detrusor end pressures were 26 cm H_2_O and 20 cm H_2_O in the first and last IUDs, respectively, and the corresponding mean bladder compliances were calculated as 9.95 mL/cm H_2_O (range: 0.034–60.0) and 16.8 mL/cm H_2_O (range: 3.0–124.3) (Figure 2).

**Table 2:** Comparison of data from first and last invasive video urodynamics (IUD) for 54 patients on whom multiple IUDs were performed.

	**First IUD**	**Last IUD**	***p*** **-value**
Age (years)	3.2 (0–12)	8.4 (2–16)	
Bladder capacity (O/E)	0.85 (0.3–1.58)	0.8 (0.15–1.8)	ns
End pressure (cm H_2_O)	26.6 (0.6–101)	21 (2–65)	ns
DO	43		
Compliance (mL/cm H_2_O)	5.75 (0.34–88.4)	16.8 (3.0–124.3)	0.0056

*All data presented as mean (range) unless otherwise specified. DO, detrusor overactivity; ns, nonsignificant; O/E, observed/expected ratio*.

In the final IUD (or the single available IUD, if only one was performed), detrusor overactivity (DO) was observed in 11% of all patients (8/71). Vesicoureteral reflux (VUR) was also encountered in 11% of all patients (8/71). One patient with grade 5 VUR was managed with a refluxing ureterostomy due to renal impairment in the first year of life. This was closed at the age of 2 years, and a hydrodistension implantation technique (HIT)/subureteral transurethral injection (STING) procedure with Deflux® was subsequently performed. This patient is currently managed with regular botulinum toxin injections. Their last recorded bladder compliance was 7.9 mL/cm H_2_O, with an end pressure of 23 cm H_2_O.

Estimated glomerular filtration rate (eGFR) data were available for 63 patients. The results were corrected to age and weight. For patients who were tested more than once, the last result was studied. The mean age was 7.5 years (range: 2 months−16 years), and the mean eGFR value was 102 mL/min/1.73 m^2^ (range: 58–140). 94% of these patients (59/63) had normal corrected values, and 100% of patients in the transition age (> 14 years) had values above the minimum when corrected for age.

CIC was required in 82% (58/71) of all patients. This began in the first 6 months, with the necessary frequency of catheterisation ranging from 4–7 times per day. 18% (13/71) of the patients were not on regular CIC. All 13 of these had safe bladders based on their last IUD, with 38% (5/13) of this group having a sacral pathology. 10% of all patients (7/71) had a bladder drainage procedure, and their details are discussed later. One patient stopped CIC after an incident of a knotted catheter and is currently awaiting further assessment as of the time of writing.

Sixty-seven patients were at least 6 years old. At the age of 6 years, 80.5% of these patients (54/67) were on CIC, and 39% (26/67) had achieved an acceptable level of continence (“social continence,” defined as completely dry or only occasionally wet with damp patches). By their last follow-up, 82% of these patients (55/67) reported to be dry, with 63% (42/67) on CIC. The mean age at the last follow-up was 11.5 years.

Anticholinergic medications remained in use in 87.5% (62/71) of all patients. Patients with inadequate bladder emptying were started on anticholinergics once the CIC training was complete. This treatment continues long-term, and a trial to stop is only offered if two conditions are met: (1) low bladder pressure with adequate bladder emptying and (2) a safe bladder according to the IUD evaluation criteria.

Botulinum toxin (Dysport®) intradetrusor injection was required in 10/71 patients during the study period. Indications were worsening bladder urodynamic study results (low compliance, high pressure or high-grade reflux) or intractable wetting despite compliance with CIC and anticholinergics. The Dysport® dose utilized was 15 IU/kg, up to a maximum of 1000 IU. Patients were seen in the clinic 3–6 months after Dysport® injection, and non-invasive bladder function assessments (BFAs) were performed. These included uroflow study and pre- and post-bladder scanning for volumes. Two patients are on the waiting list for repeat injection. Table 3 depicts the results of the patients who received botulinum toxin injections. Assessment of the injections was tailored according to the initial indications. Patients whose wetting resolved for a period and then regressed again were offered another injection without the need for another IUD. Alternatively, patients who received this treatment for unsafe bladder were assessed with a repeat IUD after 6–12 months to determine when further injection was required. The mean number of injections was 3.4 per patient (range: 1–10). DMSA was not part of the initial management protocol – it was performed in 72% (51/71) of the patients when there was a clinical suspicion of renal injury, but it was not used for routine monitoring. Mean age at the final tests was 3.7 years (range: 0–16).

**Table 3:** Results from the subgroup (10/71) of patients who received botulinum toxin A injections.

Age at first injection (years) – mean (range)	10 (5–14)
Sex ratio (M:F)	6:4
Pre-injection UD (mean):	0.879
O/E volume	37.5
P_det_ – end fill volume (cm H_2_O) Compliance (mL/cm H_2_O)	6.53
Post-injection UD (mean):	0.73
O/E volume	31.2
P_det_ – end fill volume (cm H_2_O) Compliance (mL/cm H_2_O)	11.397
Total number of injections – mode (range)	2 (1–10)
Corrected eGFR (mL/min/1.73 m^2^) – mean (range)	99.4 (64–184)

*eGFR, estimated glomerular flow rate; O/E, observed/expected bladder capacity ratio; P_det_, detrusor pressure; UD, urodynamics*.

The results of the last DMSA scans performed on these patients showed that 15.6% (8/51) had scarring or > 5% functional discrepancy. Of these eight patients, five had eGFR <90 mL/min/1.73 m^2^. All had a safe bladder at the last IUD. This also includes one patient who moved to our catchment area from abroad at the age of 12 and is currently on conservative management; one patient who had an ileocystoplasty; one patient who has a suprapubic catheter and is awaiting a drainage procedure; a patient on repeated botulinium toxin injections, CIC and anticholinergics; and a patient who had lost follow-up for 3 years before having their treatment restarted.

Seven patients underwent a bladder drainage procedure (suprapubic catheter (SPC) *n* = 1, Mitrofanoff procedure *n* = 3, vesicostomy button *n* = 3). Indications for these included having physical difficulty performing CIC (*n* = 5), while two patients' indications were not available from the notes. Of these seven patients, one had multiple false passages from CIC and required emergency SPC insertion. Five patients had Deflux® injection for VUR. One was in association with SPC insertion, and three patients had previously received botulinum toxin injection. 18% (13/71) of all patients also had an antegrade continence enema (ACE) procedure for bowel management as a separate surgery. One had a caecostomy antegrade enema, since their appendix had been previously used in a Mitrofanoff procedure. Ileocystoplasty bladder augmentation was performed in one patient within the whole cohort. The indication was progressive chronic kidney disease with unsafe bladder despite being on CIC six times daily.

## Discussion

The main objectives of all management strategies for patients with NB should be to achieve adequate bladder capacity and social continence while preserving renal function (1). Several methods, both surgical and medical, have been developed over the last few decades to achieve these goals (2). Different strategies, including CIC and anticholinergic medications, are usually involved in management. We defined a “safe” bladder as one with a detrusor pressure <40 cm H_2_O and an absence of high-grade (grade 3+) reflux. This is a reliably objective tool, but other methods have been suggested, including simple surveillance of the upper urinary tract using ultrasound (3). Despite this, a designated “safe” bladder can still require surgical intervention in 35–55% of spina bifida patients later in life, and periodic bladder assessment therefore remains invaluable (3, 10). Early assessment of spina bifida has shown that hostile bladder prevalence can be as high as 15% (2). In our cohort, this prevalence was 6%. However, since we studied patients 5 years after the start of active treatment, this improved outcome was not surprising (as patients with hostile bladders had already been on treatment). Reported bladder compliance after treatment with other methods was in fact comparable to our results, with Tanaka et al. (11) and Altaweel et al. reporting 9 and 13 mL/cm H_2_O, respectively (12).

In our previously published data, 1.6% of our patients had chronic kidney disease (CKD). This current cohort is comparable, with two patients having eGFR results below the corrected normal (13). This in turn is comparable to other studies measuring the long-term renal functions of spina bifida patients (3). We used DMSA scans to monitor renal scarring in patients with a history or US result indicating renal injury. Imamura *et al*. published data showing a strong correlation between decreased eGFR and renal scarring seen in DMSA scans. Their data suggest that this nuclear medicine scan can identify more pathological changes than eGFR, but whether routine DMSA study should be normal practice is unclear from their conclusions (14).

Since the first description of CIC in 1972 by Lapides et al. (15), the prevalence of spina bifida patients who remain on CIC for a long period of time has increased considerably. The proportion of these pediatric spina bifida patients who are routinely on CIC varies in the literature from 43–87% (10, 14). In recent recommendations from a group of experts, an early start of CIC from day 1 was deemed crucial for maintaining a safe bladder and protecting the upper urinary tract (16). This is the approach that we have been using for 20 years, but we start CIC after performing bladder function assessment in the neonatal unit to confirm that bladder emptying is affected. Within our cohort, 82% of patients were performing regular and adequate CIC. CIC is not without its limitations, however – infections are a known association (17). Six of our patients were unable to perform CIC, while one had multiple urethral false passages and acquired hypospadias. The efficacy of CIC management has also been questioned, as Woo et al. (18) suggested that early introduction of CIC did not prevent renal scars observed in DMSA scans.

Social dryness in spina bifida patients is achievable through a variety of measures. In general, it can be attained with CIC and anticholinergics alone (2). Up to 80% of urinary incontinence associated with spina bifida has been managed effectively with these conservative measures (19). 82% of our cohort was socially dry at the last follow-up, with 63% of them requiring CIC. In the decades since it was first described in the 1950s, the popularity of bladder augmentation has varied (20). The introduction of this procedure on the small, contractile bladder of spina bifida patients has made a substantial impact on quality of life (21).

However, it is widely accepted that bladder augmentation (cystoplasty) is associated with a myriad of complications, including delayed bladder rupture, adhesive bowel obstruction and bladder calculi (20). These may present later in life, most likely after the transition to adult service; therefore, they are often not handled by pediatric urologists and as such may be underreported in the pediatric literature. Cystoplasty has also been linked with malignancy, which is a major contributing factor to its fluctuating acceptance levels (22). This risk was strongly challenged by Husmann et al. (23), who published the largest dataset to date on the link between cystoplasty and malignancy. One patient in our cohort (1.6%) had bladder augmentation. A hostile bladder and deteriorating renal function were the indications.

Bladder drainage becomes increasingly challenging in older patients. Mitrofanoff and Monti procedures have gained acceptance as reliable surgical options (24). Difficult catheterisation for wheelchair-bound patients with a high body mass index was a common indication in our patients.

The success of NB management is measured by adequate growth of a safe bladder. In our experience, our management protocol increased both bladder capacity and compliance while maintaining safe pressure. A significant increase in compliance and bladder capacity resulting from botulinum toxin A injection was similarly noted by Horst et al. (25). Experts have recommended a the use of a detailed protocol with escalation of management from the less to the more invasive treatment. It is possible thus to avoid or delay bladder augmentation by following a strict treatment protocol (16). These studies and recommendations echo our experience, and we consequently postulate that our results in achieving a safe bladder are multifactorial and are a product of marginal gains from utilization of all of the less invasive methods we have described.

We used Dysport® injections in 10 patients. Repeated injections were based on the response to the initial injection and the degree to which its effects had worn off, as assessed at the follow-up. Botulinum toxin injection is an established treatment to rapidly reduce bladder overactivity, with compliance increasing up to 121% in some studies (26–28). The dose used varies within the literature, but recurrent injection is strongly linked with maintaining good cystometric capacity and reducing detrusor overactivity, as demonstrated in a study by Naqvi et al. (29). In a multicentre study by Hascoet et al. (30), Botox injection was also performed for urgency and recurrent UTIs. We used botulinum toxin injections in cases of wetting despite CIC but not in cases of urgency.

This study is limited by its retrospective nature, reliance on patients' own assessments of what defines social dryness, and deficiency of data on the appearance of the bladder neck in IUDs (these data were thus removed from the analysis). There was deviation from local guidelines based on the surgeon's decision in some cases where repeat IUD was not performed according to the protocol, and this has affected the comparison. However, this did not adversely affect the aim, since the majority of the patients did not require major surgical intervention and their renal assessment was performed adequately.

## Conclusion

A conservative strategy with regular assessment of bladder functions is a safe and replicable approach for management of neuropathic bladder secondary to spina bifida. This strategy can eliminate or delay the need for bladder augmentation in achieving safe bladder, renal protection and an acceptable level of continence.

## Data availability statement

The raw data supporting the conclusions of this article will be made available by the authors, without undue reservation.

## Ethics statement

Ethical review and approval was not required for the study on human participants in accordance with the local legislation and institutional requirements. Written informed consent from the participants' legal guardian/next of kin was not required to participate in this study in accordance with the national legislation and the institutional requirements.

## Author contributions

HE: data collection, data analysis, and manuscript drafting, TA: manuscript drafting, manuscript revision, and critical revision. KE: critical revision, FM: study design, critical revision, and manuscript review. All authors contributed to the study conception and design and read and approved the final manuscript.

## Conflict of interest

The authors declare that the research was conducted in the absence of any commercial or financial relationships that could be construed as a potential conflict of interest.

## Publisher's note

All claims expressed in this article are solely those of the authors and do not necessarily represent those of their affiliated organizations, or those of the publisher, the editors and the reviewers. Any product that may be evaluated in this article, or claim that may be made by its manufacturer, is not guaranteed or endorsed by the publisher.

## Abbreviations

ACE: antegrade continence enema
BFA: bladder function assessment
CIC: clean intermittent catheterisation
CKD: chronic kidney disease
DMSA: dimercaptosuccinic acid scan
DO: detrusor muscle overactivity
DTPA: diethylenetriaminepentaacetic acid
Egfr: estimated glomerular filtration rate
HIT: hydrodistension implantation technique
IUD: invasive video urodynamics
NB: neuropathic bladder
O/E: observed/expected
P_det_: detrusor pressure
SPC: suprapubic catheter
STING: subureteral transurethral injection
UD: urodynamics
UDS: urodynamic studies
US: ultrasound
UTI: urinary tract infection
VUR: vesicoureteral reflux.
